# The lateral habenula as a master regulator of innate and learned social behaviors

**DOI:** 10.1007/s00213-025-06911-x

**Published:** 2025-10-20

**Authors:** Grace Dodis, Sanghoon Choi, Meghan Flanigan

**Affiliations:** Department of Neuroscience, Medical University of South Carolina, 70 President Street, Rm 210, Charleston, SC 29425 USA

**Keywords:** Lateral habenula, Social behaviors, Sex differences, Antisocial, Prosocial

## Abstract

The lateral habenula (LHb) is classically associated with processing aversive stimuli and suppressing reward-driven behavior. Recent work, however, redefines the LHb as a node that not only mediates antisocial and avoidant behaviors but also regulates prosocial engagement and social motivation. As a convergence point for forebrain inputs and a driver of midbrain output, we review how the LHb contributes to behavioral outcomes in aggression, social avoidance, caregiving, and social memory. Cell-type-specific investigations reveal that glutamatergic (vGlut2⁺) and GABAergic (GAD2⁺) LHb neurons play dissociable roles in shaping aggression, social avoidance, and social cognition, while serotonin 2c receptor (5-HT_2C_) expressing neurons may represent a molecularly distinct sub-population differentially influencing social behaviors in males and females. These findings suggest that LHb circuits encode the valence and salience of social cues and flexibly adjust behavioral output in contextually appropriate and adaptive ways. Disruption of these pathways from stress, early adversity, or genetic susceptibility may underlie rigid or maladaptive social phenotypes. By mapping the input-output architecture and functional diversity of LHb subcircuits, future studies incorporating specific cell types and circuits will further unravel the complicated nature of how social decisions are dynamically regulated in the LHb and how dysregulated LHb activity may contribute to social symptoms of psychiatric disease.

## Introduction

**Fig. 1:**
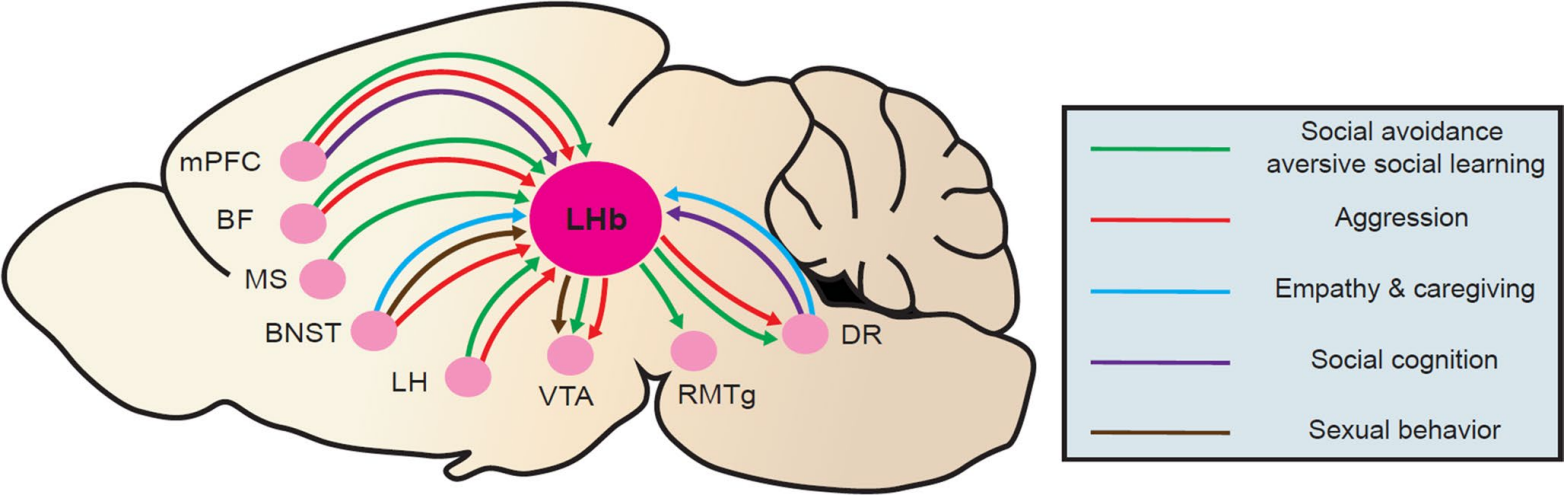
Circuit-level inputs to and outputs from the LHb organized by social behavioral domains. Summary of anatomically verified afferent and efferent connections to and from the LHb, organized by functional relevance to specific social behavioral categories. Lines and arrows are color-coded based on behavioral domains: green, social avoidance/aversive social learning; red, aggression; blue, caregiving and empathic behavior; purple, social discrimination/novelty; and brown, sexual behavior. Brain regions follow: lateral hypothalamus (LH), basal forebrain (BF), medial prefrontal cortex (mPFC), bed nucleus of the stria terminalis (BNST), pituitary gland (PG), rostromedial tegmental nucleus (RMTg), ventral tegmental area (VTA), and dorsal raphe (DR)

The lateral habenula (LHb) is a small epithalamic sub-nucleus classically implicated in negative prediction error and responses to aversive stimuli. Particularly in the past 10 years, our understanding of its role in behavior has expanded, such that it is now viewed as a critical regulator of affective, motivational, cognitive, and homeostatic processes that influences behaviors ranging from alcohol consumption to fear learning. Further, its dysfunction is strongly associated with a host of neuropsychiatric disorders, most notably major depression (MD) (Hu et al. 2020). Anatomically, the LHb receives inputs from largely forebrain nuclei, particularly the medial prefrontal cortex (mPFC), basal forebrain (BF), bed nucleus of the stria terminalis (BNST), and lateral hypothalamus (LH), while its outputs target predominantly mid- and hind-brain nuclei such as the dorsal raphe (DR), the ventral tegmental area (VTA), and the rostromedial tegmental nucleus (RMTg) (Zahm and Root 2017) (Fig. 1). However, the LHb is not merely a relay center between the forebrain and posterior structures; rather, it is a region that, through its dynamic physiological regulation by a variety of neuro-active signaling molecules and downstream control of some of the brain’s most critical neuromodulatory centers, promotes adaptive responses to an extraordinary diversity of stimuli.

Social behaviors can be divided into antisocial or prosocial categories. While both serving to increase the likelihood of survival, antisocial behaviors, such as aggression or aversive social learning, are typically exhibited in response to perceived threat and aid in protection of the animal and it’s peers along with their territory and resources. Prosocial behaviors, including caregiving and affiliative behaviors, occur within and between social networks to increase sharing of resources, cooperation, social support, and survivability of offspring. Both anti- and prosocial behaviors are highly varied both within and among species, requiring a complex interplay between multiple streams of internal and external information, followed by action sequences that can be quickly updated and modulated with changing circumstances. Given its unique ability to coordinate affective, motivational, cognitive, and homeostatic states, it may be no surprise that the LHb has now been implicated in almost every aspect of social behavior, including social play in adolescents, intermale aggression, sexual behavior, maternal caregiving, social cognition, social development, social hierarchy, and responses to social stress (Ogawa and Parhar 2022; Flanigan et al. 2017; Cobb-Lewis et al. 2024; Flanigan et al. 2023; Lecca et al. 2023; van Kerkhof et al. 2013; Wang et al. 2021). However, our current understanding of the precise mechanisms by which the LHb functionally regulates these numerous social behaviors is in its infancy.

In this review, we hope to provide a comprehensive synthesis of what is currently known about how the LHb guides social behaviors across species and sexes. While most findings discussed draw from rodent studies, we incorporate relevant human, non-human primate, or other species whenever possible. We further discuss the discrete circuits, cells, and molecular mechanisms that are currently thought to be involved and the most pressing future directions for research in this area. Contrary to what may be presumed by the classical understanding of the LHb as a mediator of behavioral aversion, we hope to illustrate that it in fact shapes flexible responding across a broad spectrum of pro- and antisocial behaviors.

## LHb anatomy and physiology

LHb anatomy is highly unique, both in its afferent and efferent connectivity and its microcircuitry. Its afferents drive complex interactions of GABAergic, glutamatergic, neuropeptidergic, and neuromodulatory signals that both influence LHb output and finely tune the activity other LHb afferents. Notably, these afferents are known to activate signaling cascades within the LHb that are highly implicated in social behavior across the lifespan, particularly vasopressin, estrogen, kisspeptin, serotonin, and dopamine. Unfortunately, only a small number of these socially-implicated signaling systems have been functionally investigated in the LHb, highlighting a necessary area for future research. In terms of outputs, LHb projection neurons send glutamatergic output signals to robustly regulate the activity of downstream dopamine, norepinephrine, and serotonin centers that project widely throughout the brain, granting it an unparalleled level of descending monoaminergic control.

**Fig. 2:**
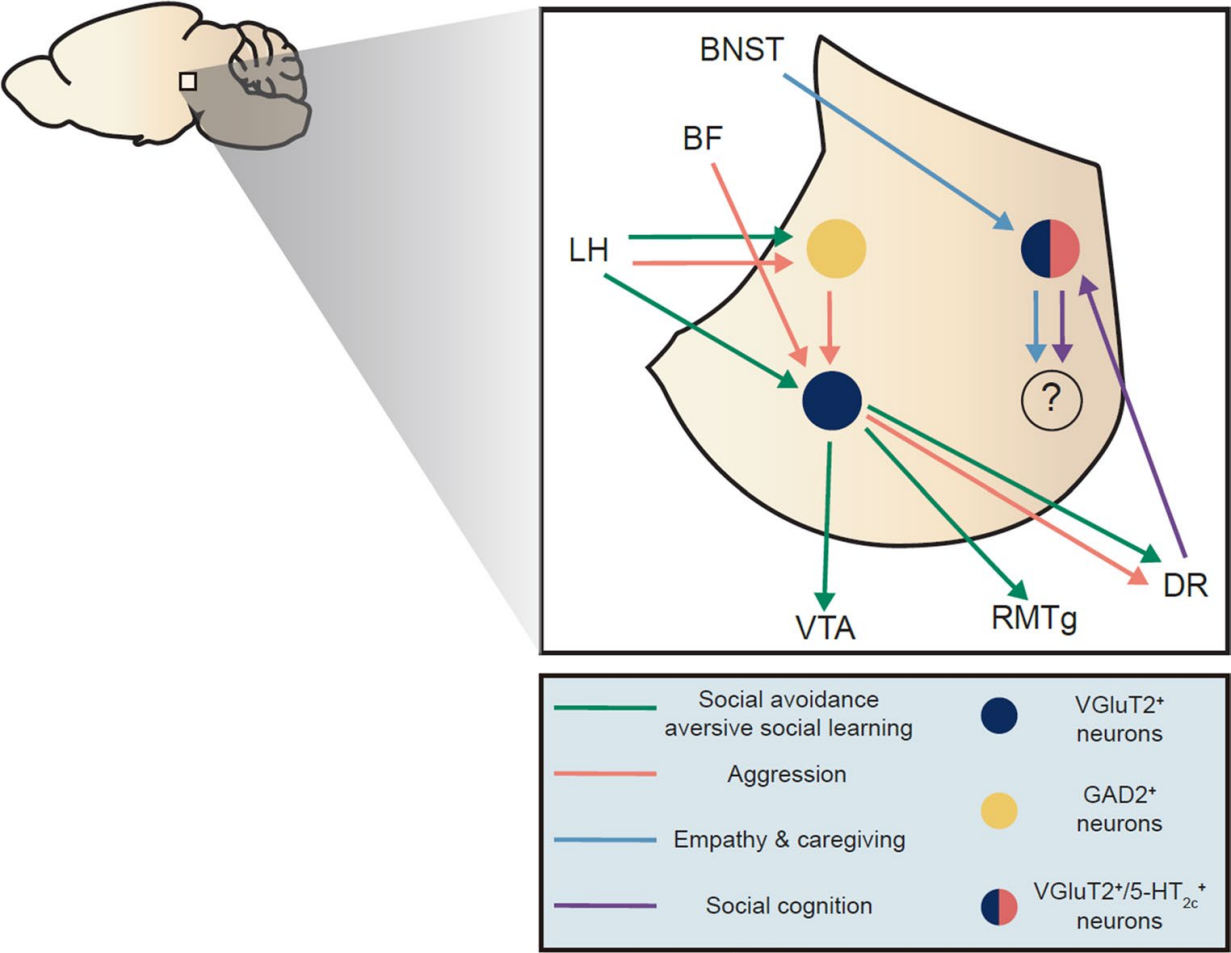
Efferent and afferent projections involving identified LHb neuronal subtypes organized by social behavioral domains. LHb afferent and efferent projections originating from molecularly defined neuronal subtypes: vGlut2⁺ glutamatergic neurons, GAD2⁺ GABAergic neurons, and 5-HT2C receptor-expressing neurons. Lines and arrows are color-coded based on behavioral domains: green, social avoidance/aversive social learning; red, aggression; blue, caregiving and empathic behavior; purple, social discrimination/novelty; and brown, sexual behavior. Connections illustrated include the dorsal raphe (DR), ventral tegmental area (VTA), lateral hypothalamus (LH) rostromedial tegmental nucleus (RMTg). Previous studies support the relevance of 5-HT2c LHb neurons with caregiving behaviors as well as social discrimination, yet their efferent projection toward other brain nuclei has not been confirmed (marked by “?”). Social behaviors that have not been investigated with respect to specific cell types within LHb are excluded from this figure (see Fig. 1 for more complete circuitry)

Glutamatergic afferents to the LHb arise primarily from the mPFC, ventral pallidum (VP), BNST, and LH, and generally drive excitation of the LHb in response to negatively valanced stimuli (Zahm and Root 2017; Huang et al. 2024; Liu et al. 2022b). GABAergic projections to the LHb arise mostly from the extended amygdala (including BNST and central amygdala (CeA), the VP, parts of the basal forebrain (e.g., the septum and diagonal band), and the LH (Zahm and Root 2017; Liu et al. 2022b). The entopeduncular nucleus, a major BF input to the LHb, co-releases GABA and glutamate to meticulously refine LHb activity (Shabel et al. 2014). In addition, serotonergic and noradrenergic innervation are provided by the DR, locus coeruleus (LC), and VTA, respectively, while neuropeptidergic modulation is provided by various subregions of the hypothalamus, including the LH (Ogawa and Parhar 2022; Bueno et al. 2019; Ren et al. 2019; Zahm and Root 2017) (Fig. 2). Serotoninergic innervation in particular provides modulation of GABA, glutamate, and co-releasing afferent activity in addition to modulating LHb target neurons directly via multiple different post-synaptic serotonin receptors (Shabel et al. 2012, 2014; Delicata et al. 2018; Xie et al. 2016; Zuo et al. 2016). While inputs from the VTA are thought to be largely glutamatergic, dopaminergic innervation of the LHb arises from the paraventricular hypothalamus (PVN) (Ariyani et al. 2025). The median raphe (MR) also provides strong glutamatergic input to the LHb, which predominantly activates LHb neurons projecting to the MR and the VTA, forming an aversive feedback loop (Szőnyi et al. 2019).

As mentioned above, outputs of the LHb have a remarkable ability to regulate the activity of monoaminergic centers in the mid- and hindbrain. While the LHb receives serotonin only from the DR, LHb outputs exert complex control over both the DR and the median raphe (MR). The LHb both directly excites serotonin neurons in the DR as well as indirectly inhibits them through di-synaptic connections with local DR inhibitory neurons (Pollak Dorocic et al. 2014; Ren et al. 2019; Okaty et al. 2020). LHb outputs to the DR have also been shown to excite non-serotonergic, vesicular glutamate-3-expressing neurons that project to the VTA (Takahashi et al. 2022). While the LHb predominantly inhibits VTA dopamine release via indirect inhibition through the RMTg, there is also a direct output pathway from the LHb to VTA dopamine and GABA neurons (Omelchenko et al. 2009; Lammel et al. 2012). In addition, the LHb activates interneurons in the laterodorsal tegmentum (LDT), which subsequently inhibits both acetylcholine and glutamatergic neurons there (Yang et al. 2016). Finally, the LHb forms yet another a recurrent feedback loop with the LC whereby LHb activation activates LC noradrenergic cells which then release norepinephrine onto LHb astrocytes, enhancing calcium signaling and further exciting LHb neurons (Bueno et al. 2019; Xin et al. 2025). These data highlight the distinctive position of the LHb as a potent regulator of dopamine, serotonin, norepinephrine, and acetylcholine in the brain.

Glutamatergic projection cells comprise the vast majority of LHb neurons and display incredible morphological, physiological, and transcriptomic diversity, showing distinct patterns of these properties in LHb subregions. While the LHb can be broken down into as many as 12 subnuclei, the majority of studies tend to distinguish LHb subregions as either medial LHb (mLHb) or lateral LHb (lLHb) (Wagner et al. 2014). The mLHb and lLHb display differences in neurotransmitter and neuropeptide receptor expression as well as innervation patterns of axons releasing ligands for these receptors. For example serotonin-positive fibers from the DR are observable primarily in the mLHb, serotonin receptor 2c (5HT2c)-containing neurons are present throughout the LHb, and dopamine receptor 2-containing neurons are in the lLHb (Aizawa et al. 2012). Single-cell sequencing characterizations of the LHb in rodents have identified up to 6 distinct transcriptional neuronal clusters displaying specific electrophysiological properties (Hashikawa et al. 2020; Wallace et al. 2020). Notably, the cell types identified in the mouse LHb were highly similar to those previously described in zebrafish, indicating a high degree of conservation between species. LHb glutamate neurons also display some anatomical segregation with respect to output projections, with the mLHb largely projecting to the DR, MR, and VTA and the lLHb largely projecting to the RMTg (Gonçalves et al. 2012). Morphologically, LHb neurons can be classified into spherical, fusiform, and polymorphous cells (Weiss and Veh 2011). They are largely categorized electrophysiologically by their resting activity patterns, which may be silent, tonically firing, irregularly firing, or burst firing (Weiss and Veh 2011). However, the vast majority of LHb neurons will burst fire when given hyperpolarizing synaptic currents, highlighting both their wide physiological dynamic range and their unique ability to display depolarization- and hyperpolarization-driven neuronal activation patterns.

While over 90% of LHb neurons express markers for the vesicular glutamate transporter-2 (vGlut2) (Hashikawa et al. 2020; Wallace et al. 2020), the idea that the LHb is devoid of local inhibition has been challenged in recent years, with multiple research groups characterizing peculiar populations of neurons expressing some, but not all, of the markers generally characteristic of GABAergic interneurons. While few neurons in the LHb express vesicular GABA transporter (vGAT), there is a sizeable population (~ 5–18%) of GABA decarboxylase-2 (GAD2) neurons that are vGAT- and vGlut2+, yet locally release GABA to inhibit LHb projection neurons (Flanigan et al. 2020; Green et al. 2023). While it remains contested, there is also evidence that some GAD2 + neurons project out of the LHb, particularly to the DR and LDTg, and release glutamate and GABA there (Park 1987; Nguyen et al. 2024; Quina et al. 2020). Given that the vast majority of GAD2 + cells lack vGAT, they may either take up and release GABA via volume transmission or vesicularly package GABA via non-canonical mechanisms to drive their local inhibitory tone, as GABA can be detected with immunofluorescence in these cells (Webster et al. 2020). Characterizing these potential non-canonical inhibitory mechanisms should certainly be a focus of future study. Perhaps one reason why these cells have been so difficult to characterize is that, like LHb vGlut2 + neurons, functional, anatomical, and transcriptomic studies have revealed a remarkable diversity among GAD2 + neurons in the LHb. Indeed, some GAD2 + clusters are defined by interneuron markers like parvalbumin and somatostatin, with each cluster exhibiting distinct physiological properties like input resistance (Webster et al. 2020). Interestingly, while parvalbumin-expressing cells in the LHb appear to exert excitatory, inhibitory, and mixed excitatory/inhibitory control over projection neurons, somatostatin-expressing cells are predominantly inhibitory in the LHb (Webster et al. 2020). This complexity of intra-LHb inhibitory connectivity is only beginning to be appreciated and will require many additional studies to fully disentangle. Despite being few in number, LHb GAD2 + neurons have been shown to play discrete roles in a variety of social and non-social behaviors, highlighting their functional relevance (Chen et al. 2024; Flanigan et al. 2020).

Altogether, there are multiple facets of LHb’s anatomy and physiology that position it as a critical switchboard for flexible, adaptive behaviors, including social behavior (Mizumori and Baker 2017; Okamoto et al. 2021). It ultimately receives streams of information from multiple inputs involved in salience detection and discrimination of social cues and transforms these inputs into motivated action sequences through its outputs to monoaminergic centers.

## Sex differences in the LHb

Within the following sections, we lay out key functions of the LHb in a variety of sex-specific social behaviors, emphasizing its functional role in intermale aggression, maternal caregiving, and female sexual behavior. We also describe sex differences in LHb regulation of social cognition, particularly social discrimination and novelty preference. In particular, LHb neurons expressing the serotonin 5HT2c receptor may represent a distinct population playing an outsized role in guiding innate higher-order social cognitive processes selectively in females. However, sex differences in LHb physiology, circuitry, and functions in the context of social behaviors are still surprisingly underexplored, leaving critical gaps in our understanding of the LHb’s role in health and disease.

Emerging evidence provides support for sexually dimorphic connectivity patterns of the LHb in both rodents and humans. For example, one rodent study reported that the female LHb receives more robust projections from the lateral septum, ventromedial hypothalamus, posterior hypothalamus, and anterior hypothalamus compared to males (Liu et al. 2022). However, it also reported that males receive more robust projections from the medial septum, which notably is a component of the BF circuit found to promote intermale aggression in the Golden et al. 2016 study discussed below (Liu et al. 2022). While another study found no overt evidence for differential targeting of the LHb by its inputs between rodent sexes, statistical clustering of afferent tracing data revealed possible unique collateralization patterns in males and females (Huang et al. 2024). Human tractography studies have also described structural sex differences in LHb, specifically that there are more overall projection fibers coming into the LHb of females than males (Hitti et al. 2022). At the output level, studies in rats have revealed that inhibition of VTA dopamine firing induced by electrical stimulation of the LHb is longer and greater in magnitude in males compared to females (Bell et al. 2023). Future studies in humans should investigate functional connectivity patterns between additional LHb inputs and outputs in both sexes, as they could reveal differences not reflected at the level of anatomical structure. These studies should be complemented by slice electrophysiology studies in rodents that interrogate potential sex differences in LHb responses to incoming neuromodulatory signals.

Regulation of the LHb by sex hormones is only beginning to be disentangled in rodent models, but existing studies suggest that estrogen and testosterone dynamically influence LHb activity and social behavioral repertoires. While the estrous cycle alone exhibits minimal alterations in electrophysiological characteristics of LHb neurons, stress exposure introduces changes in LHb excitability during the metestrus and diestrus stages, suggesting an interaction between hormonal cycles and stress exposure on female LHb neurons (Kim and Chung 2024). Female LHb neuron properties are also altered during other phases of reproduction, such as during pregnancy, parturition, and lactation (Wagner, Silverman, and Morrell 1998; Lonstein et al. 2000), indicating that the LHb may be involved in the initiation of hormonally-driven changes in behavior. While both sexes express estrogen receptors alpha and beta (ERαand ERβ) in the LHb, females show a higher density of expression, particularly in the mLHb (Bell et al. 2023). Recent studies have determined that ERs are enriched in GAD2 + neurons in the LHb, which serve to dampen LHb output through local inhibition when activated (Chen et al. 2024; Zhang et al. 2016). Accordingly, LHb cFos, a marker of neuronal activity, is reduced following administration of estradiol (Li et al. 2015). Consistent with the idea that local LHb inhibition through these neurons reduces negative affect, ER agonists in the LHb are anxiolytic in ovariectomized, but not intact, females (Liu et al. 2022). This may indicate that at baseline, female LHb activation is relatively low such that further inhibition does not impact anxiety. However, activation of ERs on LHb GAD2 + neurons lessens pain-induced anxiety-like behavior in males, while optogenetic inhibition of these neurons promotes anxiety in pain-naïve mice (Chen et al. 2024). Therefore, estrogen signaling in the LHb appears to be anxiolytic in both sexes, but its ability to relieve anxiety depends on the prior history of the individual. Interestingly, however, estrogen is not sufficient to induce maternal or sexual behaviors through the LHb (Felton et al. 1999); this could suggest that estrogen purely modulates affective changes associated with female reproductive states, or that a combination of estrogen and other hormonal or neurotransmitter signals (such as progesterone or serotonin) are required as well, and these possibilities should be investigated in future studies. Further investigating into the role of estrogen signaling in the male LHb will also be important, as its functions outside of pain and anxiety behaviors have not been explored.

Male sex hormones may appear to guide sex-specific behaviors through their actions in the LHb, though few studies to date have been performed functional experiments in this realm. Notably, neurons expressing ERs in the LHb also express ARs, which bind both testosterone and 5-alpha-dihydroxytestosterone (Zhang, Hernández, et al. 2018). Sexual activity, which raises brain testosterone levels, enhances vGAT expression in the LHb (Zhang, Hernández, et al. 2018), potentially enhancing intra-LHb inhibition in the context of sexual reward. Gonadectomy, which deprives males of testosterone, results in a near total loss of vasopressin-positive fibers in the mLHb, and these projections are restored following treatment with testosterone (Zhang, Hernández, et al. 2018). Of note, vasopressin projections to the LHb from the BNST are much denser in males compared to females, and most of this dense innervation takes place at the onset of puberty in males, when testosterone surges (Rigney et al. 2023; Rosie et al. 1993). This indicates that vasopressin signaling in the LHb is heavily influenced by testosterone signaling. At the behavioral level, activation of vasopressin receptors in the male LHb promotes social urine marking without altering social investigation, aggression, or locomotion; however, its role in female social behavior remains unclear, as no behavioral effects of pharmacological treatment in females were noted in this study (Rigney et al. 2020). Future studies should investigate whether preventing testosterone-induced development of the male BNST-LHb vasopressinergic projection functionally impacts the onset of intermale aggression and/or sexual behaviors, especially given that these events coincide with each other temporally. Together, these data indicate that the activity of the LHb is shaped by sex hormone influences in both sexes, poising it to provide necessary modulation of downstream monoaminergic centers in the context of sex-specific and sex-independent social behaviors. Going forward, studies investigating the role of the LHb in social behavior should always be mindful of integrating sex as a biological variable so that we may ultimately develop more precise treatments for neuropsychiatric disorders characterized by LHb dysfunction.

## The LHb and antisocial behaviors

The LHb’s role as a regulator of negative affective states and aversive learning underlies much of its function in modulating anti-social behavior. A quickly growing literature suggests that the LHb influences anti-social behavioral phenotypes that include aggression, social avoidance, social dominance, and aversive social learning. Importantly, neuropsychiatric conditions like MD, PTSD, and substance use disorders are characterized by both pathological changes in these anti-social behaviors and LHb dysfunction, highlighting an urgent need for understanding the precise neurobiological mechanisms within the LHb that control anti-social behaviors in both healthy and diseased states. To date, studies indicate that the LHb detects socially aversive stimuli to direct adaptive behavioral coping strategies such as avoidance or aggression, but that these strategies can become maladaptive following exposure to drugs or chronic stressors. Like its regulation of prosocial behavior, the LHb’s regulation of anti-social behavior involves social cognitive processes such as affective discrimination and social cue/context associations, as well as social memory.

### Aggression and social hierarchy

Aggression is a conserved and evolutionarily beneficial form of social behavior, serving to protect territory, establish social hierarchy, and increase likelihood of survival (Miczek 2021). Existing evidence highlights the LHb as a key region that balances information from prior social experience, threat perception, and social context to modulate the initiation, intensity, frequency, and type of aggressive behavior. This literature suggests that inhibitory inputs from the BF, orexinergic inputs from the LH, local inhibition within the LHb, and excitatory outputs to the DR are involved in modulating aggressive behaviors and their valence.

It has been largely accepted that the majority (~ 90%) of LHb neurons are excited by aversive social stimuli such as attack by an aggressor or social defeat (Lecca et al. 2017; Wang et al. 2017b; Congiu et al. 2019). Conversely, LHb inhibition is associated with reward-related stimuli (Lalive et al. 2022). In line with the functions of opposing LHb activity in aversion or reward processing, aggressive behavior can be split into a reactive, aversive type associated with increased LHb activity and an appetitive, rewarding type corresponding to dampened LHb activity (Golden et al. 2016; Golden et al. 2017; Takahashi et al. 2022; Miczek and de Boer 2005). To study rewarding or aversive properties of aggression, the classic conditioned place preference (CPP) paradigm was adapted to investigate an animal’s preference for an aggression-paired context in which the test mouse has the option to interact with a subordinate intruder animal. Test mice are pre-screened for unconditioned aggression levels and defined as non-aggressors, variable aggressors, or aggressors based on number of days in which attack is initiated after placing an intruder mouse into their home cage. Aggressors, who reliably attacked intruders with short (~ 5 s) latencies, consistently show a preference for the aggression-paired context, while non-aggressors display a conditioned place aversion (CPA) to the aggression-paired context (Golden et al. 2016). This indicates that in highly aggressive individuals, engagement in aggressive encounters is positively reinforcing. In support of this idea, aggression increases accumbal dopamine (van Erp and Miczek 2000) and optogenetic stimulation of VTA dopamine neurons enhances intermale aggression (Yu et al. 2014). Given that the LHb is a potent inhibitor of VTA DA neurons, it is well positioned to modulate the activity of these neurons to instruct aggressive encounters and their valence. Accordingly, broad optogenetic inhibition of the LHb enhances aggression and aggression CPP, while excitation suppresses these behaviors (Golden et al. 2016).

Inputs from the BF and the LH to the LHb are two key projections that modulate aggression and its associated rewarding properties through overall dampening of LHb activity. Aggression preferring animals exhibit heightened activity in the BF, which sends GABAergic projections to inhibit vGlut2-containing LHb neurons to prevent them from delivering excitatory signals to downstream targets, ultimately leading to increased appetitive aggression (Golden et al. 2016). Due to the reinforcing nature of this proactive aggression, we can speculate that downstream targets of the LHb that are important for this behavior likely include the VTA and RMTg. Non-aggressive mice that develop aversions to the intruder-paired context, however, show decreased BF activity, leading to increased LHb activation. Importantly, while manipulation of BF-LHb projections in pre-determined aggressors did increase or decrease aggression frequency and CPP scores, activating these projections in non-aggressors failed to make the animals exhibit an aggressive phenotype. This raises a key point that the LHb seems to modulate the valence of aggression but not its initiation.

LH orexin projections to the LHb modulate its activity and aggression valence through an indirect inhibitory pathway within the LHb (Flanigan et al. 2020). Stimulation of the LH-LHb orexin projection increases aggression frequency and CPP scores in pre-determined aggressors but does not initiate aggression in mice that are non-aggressive at baseline. This effect is mediated by activation of orexin receptor 2 (OxR2) present on GAD2-expressing neurons in the LHb, which then locally inhibit LHb projection neurons to reduce LHb output and amplify aggression along with its reward value. Inhibition of LH-LHb orexin neurons or LHb GAD2 + neurons conversely decreases aggression frequency and its rewarding properties by removing the inhibitory effect on LHb projection neurons. In another study, researchers found that hunger-induced potentiation of orexin neurons projecting to the zebrafish habenula promotes winning behavior, indicating that this orexin-driven aggression mechanism may be relatively conserved across species (Nakajo et al. 2020). Together, these findings indicate that inhibition of the LHb is a key mechanism driving the positive valence of aggressive contests, and that this inhibition can arise from both intra- and extra-LHb sources. Furthermore, orexin appears to be an important signal driving inhibition within the LHb to promote positive reinforcement in the context of repeated conflict wins.

In addition to modulating proactive aggression, the LHb also regulates reactive forms of aggression through its connections to the DR. Indeed, activation of LHb-DR promotes aggression in a frustration-induced aggression model, while inhibition selectively reduces this aggression subtype without affecting proactive or goal-directed forms (Takahashi et al. 2022). Interestingly, the authors demonstrated that LHb neurons promoting aggression via the DR targeted predominantly non-serotonergic, vGlut3-expressing neurons projecting to the VTA. Subsequent manipulation of this DR-VTA pathway recapitulated the effects of LHb-DR circuit manipulations, thereby identifying a di-synaptic circuit for promoting reactive aggression. Activation of DR serotonin neurons themselves interestingly had no effects on aggression. Notably these vGlut3 DR neurons drive excitation of VTA dopamine neurons, leading to dopamine efflux in the meso-accumbens reward pathway (Wang et al. 2023). As reactive aggression is generally associated with negative emotional states, engaging in aggression with a perceived threatening social target could provide some relief of these aversive emotions in a manner that is negatively reinforcing. If this is the case, then LHb activation during reactive aggression may be activating VTA dopamine neurons to drive aversive social learning, while LHb inhibition during proactive aggression may be selectively activating VTA dopamine neurons to mediate rewarding social learning. This can be evaluated in future studies examining known hot-spots for dopamine in the NAc that are involved in positive versus negative reinforcement behaviors (Kutlu et al. 2021).

Neuroimaging studies in humans displaying pathological aggression also implicate the LHb. For example, in men with intermittent explosive disorder (IED), a neuropsychiatric condition characterized by heightened irritability and impulsive outbursts of reactive aggression, LHb resting functional connectivity is altered in a manner that aligns with the rodent LHb study discussed above. In particular, men with IED exhibited greater functional connectivity between the mPFC and the LHb compared to controls, and the severity of aggressive symptoms were positively correlated to global efficiency of the LHb (Gan et al. 2019). Remarkably, a recent case study suggests that targeting the LHb for deep brain stimulation (DBS) could be an effective treatment for excessive aggression. In this study, an adult patient presenting with ASD, severe intellectual disability, drug-resistant epilepsy, and intractable aggression towards the self and others was treated with posterior hypothalamic DBS (Gouveia et al. 2021). This treatment reduced aggressive outbursts by 90% in this individual, echoing prior studies targeting the posterior hypothalamus for DBS-mediated aggression treatment. Furthermore, subsequent mapping of functional connectivity changes associated with the DBS treatment illustrated that the onset of treatment effects was associated with increased functional connectivity of the posterior hypothalamus with the LHb. Together, these studies illustrate concordance between human and animal studies and strengthens evidence for the idea that aggression is associated with aberrant LHb activity.

Aside from its role in mediating aggression, the LHb is also important for encoding and establishing social rank through its capacity to regulate motivation and mount responses to preserve environmental resources. This requires advanced social cognitive processes such as memory of social experiences, discrimination of specific individuals, and sensory assessment of the physical dominance of the social threat. In both mice and zebrafish, the LHb encodes past intermale confrontations (either as the aggressor or the attacked) to guide future dominance behaviors, allowing individuals to take the most adaptive strategy in a given context. On the one hand, individuals who have experienced consecutive wins would be motivated to engage in future contests so that they may gain more resources. On the other hand, individuals who have experienced consecutive losses would be motivated to avoid or not fight back in future contests so that they may protect their physical safety and chances of survival. In mice, repeated winning induces plasticity within the LHb such that activation during subsequent contests is greatly reduced (Flanigan et al. 2020). The vHb, the zebrafish homologue of the LHb, is activated by both escalating levels of danger or cues signaling it (Amo et al. 2014), and this process is engaged in social conflict as well. Inhibition of the vHb, while not conferring the ability to win, reduced submissive behaviors in experimental fish and caused them to approach the known dominant competitor and be attacked, and this was mediated via connections to downstream raphe neurons (Chou et al. 2016). Furthermore, forced social status loss in a non-aggressive dominance test (tube test) induces a reward prediction error-like signal in the LHb that is driven by LH-LHb induction of LHb burst firing(Fan et al. 2023). This increase in burst firing produces reductions in cortical network activity during subsequent dominance contests, ultimately promoting voluntary loss to subordinate competitors and inducing depression-like behaviors. These data indicate that the LHb is required for integrating memory of prior experiences to flexibly control whether it is more adaptive to exhibit dominant or submissive behaviors in subsequent conflicts.

In addition to the LH, BF, and DR, the LHb also has known connections to other regions implicated in aggression such as, BNST, central amygdala, periaqueductal gray, and preoptic nuclei (Zahm and Root 2017; Zhang et al. 2024a; Hashikawa et al. 2016). These various aggression loci may interact with each other in a careful balance to modulate the expression of aggression, potentially through their interactions with the LHb. Future studies should continue to uncover this circuitry and work towards an enhanced understanding of aggressive behavior, ultimately aiding in the improvement of available treatments for maladaptive aggression.

### Social avoidance and aversive social learning

The LHb has long been characterized by its role in aversive learning and behavioral inhibition, particularly through its capacity to encode negative prediction errors and inhibit reward-related systems (Mondoloni, Mameli, and Congiu 2022). Rather than simply suppressing action in response to punishment, the LHb dynamically mediates the evaluation of outcomes, integrating expectation, context, and memory to drive adaptive avoidance. These computations are central to negative reinforcement—a form of learning in which behaviors are shaped by the motivation to remove or avoid an innately or conditioned aversive stimulus.

The LHb plays an important role in the regulation of innate social preference and avoidance by balancing the positive and negative valence associated with social encounters. At the circuit level, both discrete inputs to the LHb as well as discrete LHb outputs have been implicated in this behavior. For example, stimulation of the mPFC-LHb circuit, which consists of excitatory glutamatergic pyramidal neurons, reduces social preference for a conspecific over an object in the three-chamber test by activating the LHb (Benekareddy et al. 2018). The LHb also mediates social avoidance associated with chronic opioid exposure, mounting a neuroimmune response that dampens the activity of LHb-DR neurons (Valentinova et al. 2019). Optogenetic activation of this LHb-DR pathway normalized sociability in opioid induced mice, indicating that activation of LHb microcircuits can result in either social preference (LHb-DR) or social aversion (mPFC-LHb). On a genetic level, ten-eleven translocation 2 (Tet2), an epigenetic modulator previously implicated in depressive behaviors in mice, was shown to have high expression within the LHb (Xu et al. 2023). After confirming that brain-wide *Tet2* conditional knockout resulted in reduced social interaction in mice during a three-chamber sociability test, researchers then examined whether downregulation of the protein specifically in the LHb was causing this impairment in social preference. General *Tet2* deficiency within the LHb as well as specifically within LHb glutamatergic neurons was shown to decrease social preference compared to control animals, without impacting social novelty preference. Furthermore, restoration of Tet2 rescued social preference, again without impacting social novelty preference. This suggests that Tet2 signaling in the LHb plays a role in modulating social preference without having an effect on social memory. It was further shown that Tet2 deficiency in the LHb resulted in increased neuronal activity, suggesting that Tet2 control of glutamatergic neuronal excitability may be a molecular mechanism that regulates how the LHb evaluates social information. It can be further speculated that reduced LHb Tet2 levels contribute to the pathophysiology of depression and subsequent maladaptive social behaviors seen in people with MD due to the associated increase in LHb activation.

As previously mentioned, LHb neurons exhibit increased activity during anticipation and delivery of aversive outcomes. In accordance with this, LHb activation encodes both unexpected aversive experiences (Lecca et al. 2017) as well as mediates aversive learning (Trusel et al. 2019), which respectively promote escape or avoidance, both of which serve to decrease interaction with negative stimuli or experiences. Delivery of unexpected foot shocks and aversive air-puffs both evoke escape behavior and LHb activation, suggesting that the aversiveness alone rather than the experience of pain is sufficient to drive neural and behavioral responses (Lecca et al. 2017). Projections from the LH to the LHb seem to play a key role in foot shock response, such that silencing these projections increases escape latency and reduces LHb excitation during shocks without impacting baseline activity. Interestingly, excitation of LH projections that synapse onto LHb neurons sending signals to RMTg GABAergic cells were shown to be the main drivers of aversion-guided escape behaviors. This is in line with previous data showing that exposure to aversive stimuli excited LHb-RMTg projections and that optogenetic activation of this pathway increased avoidance behaviors (Stamatakis and Stuber 2012). It can be speculated that this LHb-GABAergic neuron signaling, mediated by LH activity, might be responsible for inhibiting dopamine release in response to aversive stimuli. Recent work has further characterized distinct roles for transcriptionally-defined LH populations projecting to the LHb in aversive behaviors. For example, LH-LHb neurons expressing estrogen receptor 1 drive real-time and conditioned place aversion and induce aversive behaviors such as digging, but LH-LHb neurons expressing neuropeptide Y promote rearing behavior in an open field (Calvigioni et al. 2023). Interestingly, stimulation of medial septum (MS) glutamatergic inputs to the LHb also engender real-time place aversion, while GABAergic MS projections to the LHb counteract this effect (Zhang, Shen, et al. 2018). These roles for MS-LHb projections also extend to conditioned place aversion behaviors, indicating a role in learning. To study the LHb’s role cue avoidance learning, researchers have utilized aversive learning model(s) in which an auditory cue is paired with delivery of an aversive stimulus, allowing investigation of LHb activity in response to both the aversive stimuli and its associated cue, in addition to how this activity might drive avoidance behavior. Researchers have found that, throughout learning sessions, peak LHb activity shifts from first occurring only during the aversive stimulus to then being time matched to both cue and stimulus delivery in subsequent sessions (Wang et al. 2017a; Trusel et al. 2019; Lazaridis et al. 2019). The transition of this monophasic to biphasic LHb activation during avoidance learning seems to be driven by transient synaptic potentiation of excitatory synapses within the LHb, shown by an increase in AMPAR/NMDAR ratios after the second training session (Trusel et al. 2019). This increase is positively correlated with successful avoidance behavior and was not present in control mice or after later sessions, suggesting that AMPAR potentiation is specific to the acquisition of avoidance learning. Further research has indicated that the potentiation of LH-LHb glutamatergic synapses are critical for these effects (Lazaridis et al. 2019). Taken together, these studies suggest that the LHb is responsible for not only promoting escape behavior following the presentation of an innately aversive stimulus but also plays a key role in regulating cued and contextual learning associated with repetitive aversive experiences, a process that is crucial for the development of avoidant behavior.

The behavioral avoidance and correspondent LHb activity outlined thus far can be applied to social situations, particularly in the context of neuropsychiatric disorders and negative social experiences such as social defeat. Chronic social defeat stress (CSDS) paradigms are commonly used both to investigate the effects of social stressors and as a way to induce a depressive phenotype in rodents characterized by behavioral changes such as anhedonia and avoidance, as well as molecular adaptations including activation of the pituitary-adrenal axis (Golden et al. 2011). The standardized protocol involves repeated interaction between a C57BL/6J mouse and a larger more aggressive CD-1 mouse, with each interaction lasting 5–10 min. Between the 10 repeated sessions, the test mouse and aggressor mouse are separated only by a clear perforated divider to allow for continued psychosocial stress even after cessation of active attack. Additionally, the CD-1 mice are pre-screened for aggressive behavior to ensure consistent attack and dominance over the subordinate mouse. This paradigm reliably produces a majority of animals that are susceptible to social avoidance, with a subgroup of ‘resilient’ animals that do not exhibit social avoidance (Golden et al. 2011). Upon further investigation of susceptible and resilient phenotypes, it was found that susceptible mice exhibited CSDS-induced potentiation of LHb-DR synapses shown by an increase in AMPAR/NMDAR ratio, but resilient mice did not show this synaptic change (Silva et al. 2024). Researchers also investigated CSDS-induced changes in the LHb’s regulation of dopaminergic signaling within the VTA and RMTg of resilient and susceptible mice. In the VTA of susceptible but not resilient or control mice, it was found that CSDS induced a significant postsynaptic depression of LHb neurons as indicated by a decrease in AMPAR/NMDAR ratio. LHb-RMTg projections did not exhibit potentiation or depression in either resilient or susceptible mice, but both phenotypes showed a decrease in paired pulse ratio, suggesting presynaptic alterations. Together, this data shows that CSDS induces differing alterations in subpopulations of LHb neurons based on both the specific CSDS-induced behavioral phenotype and the particular LHb target. Knowing that CSDS results in potentiation of LHb-DR synapses, serotonin’s high implication in the modulation of approach or avoidance behaviors makes sense. Serotonin has particularly been shown to play a role in the acquisition of social avoidance after CSDS (Challis et al. 2013). Specifically, researchers found that mice susceptible to social defeat had increased activation of GABAergic cells in the DR, which corresponded with heightened inhibition of DR 5-HT neurons. Because of the known connections between the LHb and DR (Pollack Dorocic et al. 2014, Ren et al. 2019; Okaty et al. 2020), it is likely that the LHb plays a role in modulation of these defeat-induced changes in GABAergic and serotonergic DR neurons. We also know that LHb activity is increased in susceptible mice after CSDS when in proximity of the aggressor mouse and that activation of the LHb during social defeat induces a susceptible phenotype (Zhukovskaya et al. 2024). Taken together, communication between the LHb and DR seems to play a role in the acquisition of social avoidance, such that social defeat increases the activity of LHb projections synapsing onto GABAergic DR neurons, which then locally inhibit 5-HT DR neurons to decrease overall serotonin release from the DR and promote social avoidance behaviors.

In addition to the serotonergic system, orexinergic signaling also appears to play a role in modulating social defeat-induced maladaptive behavior in the LHb (Wang et al. 2021). Specifically, mice that were subjected to the CSDS paradigm displayed strong activation of orexinergic LH neurons that project onto glutamatergic LHb neurons, allowing for overall increased LHb activity representative of aversive experience. OxR2 was also shown to be co-localized with Fos-positive glutamatergic LHb neurons following social defeat, indicating that the LHb neurons that were activated due to social defeat may have been so due to orexin being released into the LHb and activating OxR2 on LHb glutamatergic cells. When orexin A was administered directly into the LHb of stressed mice, social avoidance behaviors were significantly decreased. Optogenetic activation of orexinergic LH neurons was also shown to decrease social avoidance following CSDS. Orexinergic projections from the LH to the LHb have been previously discussed in this review for their regulation of aggression and its associated reward value (Flanigan et al. 2020), so their implication in resilience to depression and anxiety-like behaviors following social defeat during aggressive encounters is unsurprising and fits with the overarching theme that the LHb balances information from both rewarding and aversive experiences to promote or repress learned social associations.

Another way to investigate social avoidance behaviors, more in the context of social anxiety disorder rather than MD, is through a social fear conditioning paradigm (Toth et al. 2012). Subject mice are placed in a chamber and are allowed to freely explore a caged stimulus mouse or an empty cage (Tian et al. 2024). During a 20-minute conditioning session, the experimental mouse receives a foot shock during any investigation of the stimulus mouse. The experimental mouse then undergoes a social preference-avoidance test 24 h later. In addition to conditioned mice exhibiting decreased social approaches, shorter investigations, and overall, more avoidance behaviors in the preference-avoidance test, researchers also discovered a projection from the medial prefrontal cortex (mPFC) to the LHb that showed a robust increase in Ca^2+^ signaling when conditioned mice encountered the social stimulus mouse. Neither the avoidance behaviors nor the increased mPFC-LHb Ca^2+^ signaling were present in unconditioned mice, suggesting these changes occurred due to the acquisition of social fear learning. Optogenetic activation of these fibers in naïve mice increased social fear and avoidance behaviors without inducing place avoidance, while inhibition of the same projections in mice that underwent social fear conditioning decreased social avoidance. Further, it was found that LHb neurons themselves also had increased activity during the social preference-avoidance task when conditioned mice, but not unconditioned mice, interacted with the social stimulus. This indicates that the mPFC-LHb circuit, and the LHb more broadly, plays a key role in mediating aversive social learning. In addition to glutamatergic inputs from the mPFC, excitatory projections from the BF have also been shown to mediate avoidance behaviors after social fear conditioning (Wang et al. 2024). Similarly to mPFC projections, BF projections show robust activation when a mouse encounters a stimulus mouse after conditioning, and this activation corresponds with social fear and avoidance behaviors shown in the three-chamber social interaction test. Again, inhibition of these fibers decreased these behaviors in conditioned mice. This data, combined with data from Tian et al. 2024, shows that the LHb receives multiple sources of glutamatergic input that seem to converge to carefully regulate socially conditioned fear and avoidance responses.

Remarkably, the LHb appears to undergo robust developmental shifts in activity and neuromodulation that flexibly regulate social avoidance and approach behavior depending not only on age, but rearing conditions and the presence of ambient predator threats. A recent study revealed that in typically-reared rats, the LHb transitions from low to high levels of tonic firing and bursting between infant and juvenile ages, which is associated with concomitant decreases in 5HT2c, ERα, and GABA_B_ expression (Cobb-Lewis et al. 2024). Interestingly, early care adversity (ECA) during infancy quickly altered LHb infant firing patterns to resemble those of juveniles, particularly that infants displayed increased tonic bursting behavior. In juveniles, ECA led to increased bursting, increased 5HT2c receptor expression, and decreased Erα expression, indicating that developmental patterns of LHb activity and neuromodulation can be dysregulated by ECA. The authors next chemogenetically inhibited LHb activity in infants and juveniles during sociability tests after they had experienced standard rearing or ECA. While ECA reduced social approach in infants, chemogenetic inhibition of the LHb did not alter social approach in either infants or juveniles regardless of rearing condition. However, when infants were tested for social approach in the presence of ambient threat (predator odor), LHb inhibition suppressed social approach in standard reared, but not ECA rats. Conversely, LHb inhibition in juveniles increased social approach under ambient threat conditions. Of note, the LHb cells that were activated by social testing under conditions of threat in infants versus juveniles were distinct LHb populations; it would be informative in the future to evaluate potential transcriptional markers identifying them. Given that broad chemogenetic inhibition was not able to modulate infant social suppression following ECA, the authors performed a circuit-specific manipulation experiment chemogenetically inhibiting the mPFC-LHb pathway. This selectively rescued social approach behavior in ECA infant rats, but did not impact behavior in standard reared infants, standard reared juveniles, or ECA juveniles. Taken together, these data paint an intricate picture of the LHb’s role in development, social approach and avoidance, and responses to stress. First, LHb activity and neuromodulation by serotonin and estrogen is dynamically regulated during development and sensitive to ECA. Second, the manner in which the infant and juvenile LHb encodes and mediates social approach behavior is fundamentally different, with LHb activation driving social approach in infants and inhibiting it in in juveniles. Finally, the mPFC-LHb pathway is critical for promoting social avoidance behavior associated with ECA and ambient threat exposure in infants, perhaps suggesting that ECA accelerates development of these socially-sensitive pathways to dysregulate behavior long-term.

Overall, the LHb is a major regulator of both innate conditioned social avoidance behaviors. It quickly assesses contextual information of perceived danger to promote behaviorally adaptive responses, with repetition of aversive experiences inducing potentiation of the LHb to promote conditioned avoidance. Furthermore, the LHb is important for regulating flexible social avoidance behavior across the lifespan in conditions of both acute and chronic stress, with key differences in how its activity modulates behavioral responses to social targets from infancy to adulthood.

## The LHb and prosocial behaviors

Despite its usual association with aversive responses and anti-social behaviors, there is growing evidence from primarily rodent studies that the LHb contributes to a range of prosocial behaviors. While our conception of the LHb as a driver of prosocial behaviors is not well defined at a molecular or circuit level, current evidence suggests that it may be particularly important for prosocial behaviors that are motivated by a need to correct socially aversive states, such as those induced by social isolation or others in distress.

### Maternal and caregiving behaviors

The LHb receives inputs from multiple brain regions implicated in maternal care and motivation, including the medial and lateral preoptic nuclei of the hypothalamus, the BNST, and the DR (Zahm and Root 2017; Liu et al. 2025). While only a small literature exists on the role of the LHb in maternal behavior, these studies support the idea that the LHb plays a pivotal role. For example, multiple findings illustrate that the LHb is required for the onset of maternal behavior in rodent dams, including pup retrieval, nest building, and nursing, as well as the initiation of postpartum estrous (Matthews-Felton et al. 1995; Benedict et al. 2025; Corodimas et al. 1995). This suggests that the LHb is hormonally responsive following parturition. Interestingly, the role of the LHb in maternal behavior is not limited to sexually or maternally experienced females. Lesions of the LHb also prevent maternal behavior in virgin females, in which caregiving can usually be generated with repeated exposure to pups (Felton et al. 1998). Thus, the LHb serves as both a hormonally-responsive and experience-dependent regulator of maternal behavior.

Recently, more advanced neuroscientific techniques like microendoscopic calcium imaging have enabled us to gain a more refined understanding of the encoding of pup signals in the female LHb and how those signals guide caregiving behavior. In a fascinating recent study by the Mameli laboratory, Lecca et al. found that the adult female LHb encodes pup distress signals in mice, which primarily arise from pup vocalizations when they are separated from their mother. The aversive nature of pup cries activates subsets of LHb neurons in the medial subdivision, which subsequently promote increased maternal motivation to retrieve scattered pups (Lecca et al. 2023). Glutamatergic inputs from the BNST are critical for this pup-call-induced activation of the LHb, and these inputs predominantly drive activation of postsynaptic LHb neurons in the mLHb. Optogenetic activation of the BNST-LHb pathway promoted pup retrieval in virgin females, while suppressing it in mothers greatly dampened pup retrieval. This indicates that there may be a select population of LHb neurons that promote prosocial motivation by receiving aversive social information from upstream social detector regions like the BNST. Subsequent single-cell sequencing of LHb neurons activated by pup calls demonstrated that pup-responsive LHb neurons are enriched in the serotonin receptor 5HT2c, and that this transcriptional identity is conserved across females whether they are mothers or virgins. However, whether the 5HT2c receptor itself plays a role in regulating maternal behaviors in the LHb remains unknown and should be investigated in future studies. In a follow-up study, these same authors found no evidence for plasticity in LHb neurons following exposure to pups in virgin males or females, indicating that the LHb could be an innately-programmed encoding system when it comes to caregiving behaviors in females (Wu et al. 2025). While the functional role of the LHb in paternal behaviors has not been explored, pup exposure in paternally experienced California mice, which exhibit caregiving behavior similar to female California mice, induces cFos in the LHb as well as the caudal DR (De Jong et al. 2010). Given that the caudal DR contains neurons projecting to the LHb (Flanigan et al. 2023), this potentially implicates serotonin-driven activation of the LHb in paternal behavior. Moreover, these results indicate that the LHb may be involved in caretaking behavior in both sexes depending on the innate and experience-dependent parental behaviors of the species/strain. Future studies should aim to translate these preclinical findings to humans, aiming to determine whether the LHb undergoes structural or functional changes in pregnancy or postpartum and if the LHb is activated by aversive offspring-associated stimuli in mothers and fathers.

### Social affiliation and prosocial bonding

Akin to its role in maternal and caregiving behaviors, the LHb’s role in social affiliation and bonding hinges on its ability to encode information about aversive social environments and promote adaptive prosocial responses. One context in which it appears to do this is during the induction of adolescent social play behavior. Brief social isolation in adolescent rats promotes a rebound of social play behavior when isolated animals are re-grouped, indicating an increase in social motivation following a socially-deprived state. In one notable study, social isolation was associated with enhanced LHb cFos, such that the degree of cFos activation increased as a function of the length of the isolation period (van Kerkhof et al. 2013). Re-grouping of isolated animals and engagement in social play selectively reduced cFos in mLHb neurons, indicating that the LHb is dynamically responsive to both socially aversive (isolation) and rewarding (play) experiences. The authors next performed pharmacological inactivation of the LHb and found that it reduced pinning and total play duration without altering non-play social behavior. This implies that activation of the LHb in response to the aversive isolation state is necessary for promoting adaptive social engagement in adolescents when re-introduced to a peer, much like how LHb activation in response to pup cries is required for active pup engagement in mothers. Future research in this area should aim to perform in-vivo neural recording experiments that investigate the precise patterns of LHb activity during social isolation and subsequent peer reunion in adolescents exhibiting affiliative play behavior.

The role of the LHb in social homeostasis in adults has only begun to be explored, but results in females suggest that LHb activity is dynamically modulated by inputs sensing social isolation. Similar to juveniles, brief acute social isolation in adult rodents induces a rebound of social interaction behavior when mice are reunited with their cage-mates. Using a combination of calcium imaging and Fos-based activity mapping, the authors demonstrated that glutamatergic neurons in the mPOA track isolation state in a manner that reflects both the length of isolation and predicts the strength of social rebound during reunion (Liu et al. 2025). Optogenetic stimulation of these glutamatergic mPOA “isolation” neurons enhanced social rebound after isolation, while their inhibition dampened this rebound. Subsequent activity-dependent anatomical mapping of mPOA isolation neuron outputs revealed that these cells send robust projections to the LHb that are activated during isolation, predominantly targeting LHb neurons enriched in protocadherin 10 and neuromodulin 43. However, when researchers optogenetically stimulated the mPOA-LHb isolation pathway, they were able to induce a real time place aversion without altering food intake or social rebound behavior. This suggests that while the LHb may be important for mediating negative emotional states associated with acute social isolation, it does not directly modulate social rebound. Contrary to acute social isolation, chronic social isolation is associated with long-term reductions in social interaction upon social reunion, highlighting opposing behavioral adaptations to isolation periods of different lengths. It is possible that compared to acute isolation, chronic isolation induces greater mPOA isolation neuron-induced activation of the LHb, ultimately reaching a threshold that promotes social avoidance during reunion in addition to stronger negative emotional states.

There is also evidence that the LHb may be important for mediating active empathic behavior, which at its core involves the detection of a negative social state in another individual and the initiation of prosocial approach actions to correct it. Rats will work to free a familiar conspecific from an enclosure, and the probability of a rat freeing their peer is positively associated with the strength of their affiliative interactions prior to restraint (Hazani et al. 2025). This indicates that closer social bonds may elicit more extreme aversive states when experiencing a conspecific in danger. Compared to rats that did not free their mates from the enclosure, rats that freed their mates exhibited greater cFos activation in the LHb, perhaps suggesting that the LHb serves as a substrate for evaluating the strength of prosocial bonds and guiding social decision making accordingly (Hazani et al. 2025).

While mice generally do not engage in active helping behavior, they can display affective empathy, which includes mimicry, emotional contagion, and soothing behaviors (Sivaselvachandran et al. 2018). Mice have been shown in multiple models to socially transfer stress or fear-related information to each other via multiple routes of social communication, including pheromones and vocalizations. For example, social observation of fear learning in a cage-mate will elicit conditioned responses to foot-shock-related cues in animals that did not previously receive foot-shock (Silverstein et al. 2024). Observation of cage-mates undergoing fear conditioning is associated with increased LHb activation as measured by cFos immunofluorescence, indicating that emotional contagion and/or social learning may involve the LHb (Zhang et al. 2024). Importantly, the involvement of the LHb in this form of empathy does not appear limited to peers in fearful states, but also extends to peers in painful ones, as individuals exhibiting decreased paw withdrawal threshold following a 1 h interaction with a cage-mate in a neuropathic pain state also display increased LHb cFos (Zhang et al. 2024). Under some conditions, the observer of inescapable stress may be primed to respond to future stressors with increased behavioral resilience (Mondoloni et al. 2024). The LHb also regulates this type of behavioral response; DR serotonin projections to the LHb are activated during observation of a cage-mate exposed to inescapable stress, which reduces LHb activity, in particular burst firing, to promote resilience when the stressor is directly experienced. This reduction in bursting due to increased serotonin influx appears to create a floor or occlusion effect whereby serotonin no longer modulates burst firing in the LHb, but nonetheless this induces a long-term (7d) behaviorally-resilient state. This is particularly notable because LHb bursting is thought to drive depressive-like behavior in animal models and MD in humans (Cui et al. 2019). Blocking serotonin release in the LHb during social observation of inescapable stress prevents this behavioral resilience effect, indicating its functional necessity for preventing the social transfer of acute stress. Taken together, these data indicate that the LHb can flexibly encode social observation of stress to promote either future resilience or susceptibility and that serotonin plays a key role in determining these outcomes. Future studies should investigate how the LHb computes these divergent behavioral responses depending on factors like the affiliative bond with the animal being stressed, whether there are cues associated with the stress experience, and whether the stress is escapable.

### Sexual behaviors

The LHb is interconnected with many nodes of the sexual behavioral network, including the BNST, hypothalamus, striatum, and VTA (Ogawa and Parhar 2022). In addition to expressing ERs and ARs, it also expresses progesterone receptors, oxytocin receptors (OxtR), kisspeptin receptors, and gonadotropin releasing hormone (GnRH) receptors (Ogawa and Parhar 2022). While only a handful of studies have investigated the role of the LHb in sexual behavior, the mere fact that it contains this wide variety of hypothalamic-derived sex hormone receptors indicates some involvement is likely. Notably, there is currently more support for LHb regulation of female sexual behavior than male. Lesions of the LHb do not affect male sexual behavior in rodents, but greatly reduce estrogen/progesterone-primed mating behaviors in females (Modianos, Hitt, and Flexman 1974). Furthermore, progesterone infusion directly into the LHb promotes both proceptive and receptive components of female sexual repertoires, while a progesterone antagonist reduces these behaviors (Tennent, Smith, and Davidson 1982; Etgen and Barfield 1986). This suggests the progesterone is a key hormonal signal in the LHb driving female-specific sexual behaviors. A great deal of future work remains to untangle the particular signaling molecules and circuit mechanisms mediating the LHb’s functional role in both female and male sexual behavior, but should begin with evaluating the functions of kisspeptin, oxytocin, and GnRH, which have yet to be explored in any behavioral studies.

### Social cognition

Although not widely explored in prior research, the LHb likely plays a significant role in higher order social cognitive processes, particularly those related to social memory, novelty preference, and discrimination. Prior studies have used a single test, the three chamber test, to assess these components of social cognition. In the three-chamber test for social novelty, experimental mice are given a choice between investigating a novel and familiar mouse that are separately confined to enclosures on opposite sides of the apparatus. Behaviorally naïve mice generally prefer a novel mouse over a familiar one. This preference may be interpreted as social novelty preference, but also as social memory/recognition. An active avoidance of the novel mouse generally indicates deficits in the former, while no difference in investigation between the two mice generally indicates deficits the latter. So far, current evidence suggests that while LHb activity is required for this behavior, it can also be disrupted by aberrant hyperactivity.

Bidirectional manipulations of the LHb modulate social novelty preference behaviors depending on the prior alcohol history and sex of an individual, both of which can influence baseline LHb activity. For example, in alcohol-naïve mice, chemogenetic activation of LHb neurons expressing the serotonin 5HT2c receptor (LHb_5HT2c_) induces a social novelty-averse state in males, but not females (Flanigan et al. 2023). Inhibition of these neurons in males improves social novelty preference, and this is true for naïve mice and mice with a history of binge alcohol consumption. In females, inhibition of LHb_5HT2c_ abolishes social discrimination in naïve mice such that they show no preference for a novel versus familiar mouse but normalizes deficits in social discrimination in mice with a history of binge alcohol consumption. These deficits in social discrimination associated with alcohol were also associated with increased excitability of mLHb neurons, indicating that when LHb_5HT2c_ activity is either too high (after alcohol) or too low (after chemogenetic inhibition in naïve mice) social recognition is disrupted. Remarkably, a bidirectional pattern of social recognition phenotypes in females was not observed when LHb 5HT2c receptors were genetically knocked down, such that naïve mice were unaffected but mice with a history of binge-alcohol consumption regained their social recognition abilities, suggesting alcohol led to aberrant activity of these receptors. In males, knockdown of the 5HT2c receptor did not impact social recognition or novelty preference. These results indicate a critical sex difference in the regulation of social cognition by the LHb in males and females. In females, fine-tuned levels of activity are required for appropriate social discrimination and can be shaped by serotonin 5HT2c receptors, but in males, decreasing or increasing LHb_5HT2c_ activity turns up or turns down the dial, respectively, on the positive valence of social novelty in a 5HT2c-independent manner. Taken together with findings in the realm of maternal behavior, these results suggest that LHb_5HT2c_ neurons may play a specialized role in coordinating female responses to salient social stimuli, particularly those localized to the mLHb.

In non-human primates, there is evidence that the LHb is also engaged in adaptive social memory and recognition processes. In a recent study by the Hikosaka lab, the authors illustrated that the LHb is responsive to salient social stimuli in rhesus macaques, such that it is briefly inhibited when presented with images of novel faces. The authors further used specific novel faces as cues during a reward conditioning task, illustrating that viewing of high reward value-associated faces resulted in greater LHb inhibition than viewing of low reward value-associated faces. This indicates that the LHb may be able to integrate conditioned reward associations with social stimuli to facilitate adaptive learning in primates. Interestingly, monkeys gazed at high reward-value faces for longer than low reward-value faces even though this behavior did not alter the outcomes of the task in any way, possibly suggesting enhanced reward salience when social cues are involved. Future studies should investigate whether these types of social reward conditioning tasks engage the human LHb in a similar manner. Whether the LHb is also engaged in more complex social discrimination tasks, such as those requiring discrimination of affective states in others, should be investigated in humans and non-human primates as well.

## Conclusion

In this review, we set out to comb the existing literature and provide a comprehensive synthesis of the various aspects of social behavior that the LHb is currently known to play a role in. While there exists a larger understanding for its part in regulation of negative affective states in the context of neuropsychiatric disorders, we show that there is currently a growing literature with a great deal of evidence supporting the LHb’s function in regulating adaptive social functioning by flexibly promoting either prosocial or anti-social behaviors.

To elicit antisocial behaviors such as aggression or avoidance, the LHb seems to take in information during any given social situation and bidirectionally communicate with multiple areas of the brain, including emotional, reward, hormonal, and higher cognitive centers, to induce behavior appropriate for the situation that will maximize benefit of the outcome. For example, to protect food or territory in times of threat, the LHb can become more active to drive an aversive state and promote reactive aggression. If an animal has a prior history of winning aggressive encounters, an experience that might increase access to resources and thus be considered reinforcing, their LHb activity may decrease during conflict to disinhibit reward centers and increase motivation for goal-directed aggression. Repeated or forced conflict losses, however, would increase LHb activity and promote social avoidance. Various streams of information in and out of the LHb help to modulate aggressive approach and/or social avoidance behaviors. Inputs involved in this modulation include LH orexinergic, DR serotonergic, BF GABAergic, as well as mPFC and BNST glutamatergic inputs, while relevant LHb outputs include glutamatergic projections to the RMTg, VTA, and DR. Any disruption to the careful balance of activity in these projections can result in maladaptive aggression or avoidance that is seen in many neuropsychiatric conditions, including MD, IED, anxiety disorders, and autism spectrum disorders, outlining the importance of continuing the investigation of the LHb’s involvement in these behaviors. Future work should focus on a continued effort to uncover the circuitry involved in antisocial behaviors, including the involvement of specific LHb cell types, as well as place emphasis on sex differences within this circuitry.

Outside of its more well-known functions in modulation of negative states and antisocial behavior, the LHb has an underappreciated role in regulation of prosocial behavior. Engaging in prosocial behaviors necessarily requires motivation, but this motivation may be driven by positive or negative reinforcement depending on the individuals involved and the environmental context. While the LHb largely suppresses prosocial behaviors that are motivated by positive reinforcement, it appears to play an important role in promoting prosocial behaviors that are driven by negative reinforcement by enhancing the motivation to correct a socially aversive state in the self or others. Indeed, we describe above that LHb activity is required for mothers to respond to their crying pups by carrying them back to the nest as well as for adolescents to reinforce social bonds after isolation by playing with their siblings. While the specific encoding of these negatively instructive social signals has only begun to be explored, neurons localized to the mLHb that express the serotonin receptor 5HT2c may represent a socially-tuned population that receives incoming information from social detector regions like the mPOA, the BNST, and the hypothalamus to guide sex- and developmentally-specific social behaviors. Future studies should aim to determine whether this cell population plays any role in male-specific social behaviors such as aggression, which can involve aspects of both positive and negative reinforcement. Beyond regulating prosocial behaviors involving negative reinforcement, LHb_5HT2c_ neurons have been shown to play a role in higher-order social cognitive behaviors like social novelty preference, a behavior which requires both social memory and the ability to accurately discriminate between conspecifics while also assessing the motivation to seek out novel social contacts. LHb_5HT2c_ bidirectionally modulate social novelty preference in males, inducing a robust avoidance for novel conspecifics when activated and a robust preference when inhibited. However, these neurons may function more as mediators of social recognition in females, as inhibition results in a lack of discrimination between novel and familiar conspecifics altogether. Social cognitive processes are also required for empathy, whether this empathy is expressed as active helping or emotional contagion (social observational learning). Serotonin release in the LHb robustly modulates the latter, highlighting again the multifaceted role this neuromodulator plays in multiple aspects of the LHb’s regulation of prosocial behaviors. Novel models for assessing social cognition and social motivation, such as social affective preference tests or social self-administration, respectively, will be critical for further elucidating the nuanced role that the LHb plays in prosocial interactions.

Despite many studies identifying an important role for the LHb in modulating social behaviors in preclinical models, few clinical studies have sought out to directly investigate the LHb’s contribution to human social behavior. However, a vast literature of clinical neuroimaging studies confirms structural and functional LHb abnormalities in a wide variety of disorders characterized by social dysfunction, including depression (Fortin et al. 2025), anxiety (Ma et al. 2020), substance use disorders (Oh et al. 2020), schizophrenia (Zhang et al. 2017; Xue et al. 2023), ASD (Germann et al. 2021), and IED (Gan et al. 2019). Remarkably, case studies and clinical trials evaluating the effectiveness of LHb DBS in alleviating symptoms associated with depression and schizophrenia report improvements in social functioning; however, whether this improvement is directly related to alterations in LHb activity or is merely a consequence of reduced non-social symptoms is unclear. Nevertheless, patients receiving DBS for treatment resistant depression report as much as a 35% reduction in functional social impairments (Zhang et al. 2022), while LHb DBS in a schizophrenia case study was effective in reducing aggressive behavior (Wang et al. 2020). Heightened aggression in the context of ASD may also be related to aberrant LHb activity, as DBS targeting the posterior hypothalamus, which reduces aggressive outbursts by 90%, is associated with altered functional connectivity between the posterior hypothalamus and LHb. Taken together, these studies highlight the potential promise of LHb DBS as a clinically viable treatment for social withdrawal in depression and/or heightened aggression in schizophrenia and ASD. Future studies should aim to evaluate LHb responses to positive and negative social stimuli in both healthy patients and patients with affective and neurodevelopmental disorders so that we can more deeply understand the complex role that this unique structure plays in typical and aberrant social functioning. There should also be a concerted push to collect more robust social data in studies performing LHb DBS so that we may further delineate the functional role of the LHb in discrete social processes such as social reward, social anxiety, and social cognition. The results of these studies may ultimately facilitate the development of pharmacological and/or neural stimulation approaches targeting the LHb for treating social dysfunction in a variety of patient populations.

## Data Availability

Not applicable.
